# Case Report: *De novo USP9X* missense mutation in a male fetus with pulmonary atresia and ventricular septal defect: expanding the genotype-phenotype spectrum of *USP9X*-related disorders

**DOI:** 10.3389/fcvm.2026.1726544

**Published:** 2026-03-10

**Authors:** Tingting Man, Hairui Sun, Xiaoyan Hao, Xiaowei Liu, Yihua He

**Affiliations:** 1 Maternal-Fetal Medicine Center in Fetal Heart Disease, Beijing Anzhen Hospital, Capital Medical University, Beijing, China; 2 Beijing Lab for Cardiovascular Precision Medicine, Capital Medical University, Beijing, China; 3 Key Laboratory of Maternal-Fetal Medicine in Fetal Heart Disease, Beijing, China

**Keywords:** congenital heart disease, genotype-phenotype correlation, pulmonary atresia with ventricular septal defect, *USP9X*, whole-exome sequencing

## Abstract

**Background:**

Pathogenic variants in the X-linked *USP9X* gene, which evades X-chromosome inactivation, have been predominantly linked to neurodevelopmental disorders (NDDs). Accumulating evidence has linked *USP9X* dysfunction to congenital heart disease (CHD), yet the specific genotype-phenotype correlations in this context remain poorly characterized. Pulmonary atresia with ventricular septal defect (PA/VSD) represents a severe and complex form of congenital heart disease (CHD), characterized by heterogeneous etiological mechanisms.

**Case presentation:**

A 34-year-old G2P1L1 woman was referred at 22 weeks of gestation for prenatal echocardiography due to suspected fetal cardiac anomaly. Echocardiographic evaluation identified a male fetus with PA/VSD. Following detailed counseling, the couple elected to terminate the pregnancy due to the poor prognosis and opted for subsequent genetic testing. Trio whole-exome sequencing (WES) identified a *de novo* hemizygous missense variant in *USP9X* (NM_001039590.3: c.5186A > G; p.His1729Arg; rs2147230302) in the male fetus. This variant is categorized as “Likely Pathogenic” in ClinVar for female-restricted syndromic neurodevelopmental disorders (NDDs), yet it has not been previously linked to congenital heart diseases (CHDs), specifically PA/VSD. The p.His1729Arg substitution impacts a conserved residue within the structurally critical zinc-finger domain of the *USP9X* protein. Comprehensive genetic screening failed to identify additional pathogenic variants that could explain the observed phenotype.

**Conclusion:**

This case represents the first report establishing a link between the *de novo* p.His1729Arg variant in *USP9X* and the CHD phenotype of PA/VSD in a male fetus. This finding broadens the known phenotypic spectrum of this likely pathogenic *USP9X* variant, underscoring its pleiotropic effects and implicating the gene in critical cardiac developmental pathways. Routine cardiac assessment is recommended for individuals harboring pathogenic *USP9X* variants.

## 1 Introduction

Pulmonary atresia with ventricular septal defect (PA/VSD) is among the most complex cyanotic congenital heart diseases (CHDs), defined by complete obstruction of the right ventricular outflow tract (RVOT) at the level of pulmonary valve, in conjunction with VSD (1). Its etiology is multifactorial, with established associations to 22q11.2 deletion syndrome (2), yet the majority of cases remain idiopathic. Identifying novel genetic etiologies is pivotal for unraveling disease pathogenesis and enhancing genetic counseling accuracy.

The *USP9X* gene, mapped to Xp11.4, encodes a deubiquitinase (DUB) that plays a critical role in regulating protein turnover and stability by removing ubiquitin chains (3). Crucially, *USP9X* evades X-chromosome inactivation, rendering its dosage critical for normal development (3). Pathogenic variants in *USP9X* are established causes of X-linked neurodevelopmental disorders (NDDs), including X-Linked Intellectual Developmental Disorder 99 (XLID99; OMIM #300919) (5) in males and a more complex syndromic form (MRXS99F; OMIM #300968) (4) in females, both attributed to haploinsufficiency. The recognized phenotypic spectrum encompasses intellectual disability (ID), global developmental delay (GDD), behavioral abnormalities, and structural brain anomalies (5).

While predominantly associated with NDDs, accumulating evidence indicates that *USP9X* dysfunction can also disrupt cardiac morphogenesis. Cardiac anomalies have been documented as part of the female-specific XLID99 phenotype (5). A recent report (6) further described a male individual with a *de novo USP9X* missense variant (p.Met1824Val) presenting with complex CHD, including VSD and aortic arch anomalies, in conjunction with NDD features. However, a definitive association between *USP9X* variants and the severe phenotype of PA/VSD, specifically in males, remains to be established.

Furthermore, the specific variant p.His1729Arg (rs2147230302), categorized as “Likely Pathogenic” for female-associated NDDs in the ClinVar database (VCV001338822) (9), has not been previously reported in conjunction with any CHD phenotype. Here, we report the case of a male fetus with a diagnosis of PA/VSD harboring a *de novo* hemizygous p.His1729Arg variant in *USP9X*. This report aims to broaden the known genotype-phenotype landscape of *USP9X* and furnish novel clinical evidence underpinning its role in molecular pathways governing cardiac outflow tract and septal morphogenesis.

## 2 Case presentation

A 34-year-old gravida 2, para 1, living child 1 (G2P1L1) woman underwent routine prenatal care. Her prior pregnancy resulted in a healthy full-term female infant, and her medical history was unremarkable. The early pregnancy course was uncomplicated, with the exception of a common cold. First-trimester screening, including Non-Invasive Prenatal Testing (NIPT), revealed low-risk results for common aneuploidies.

**Figure 1:**
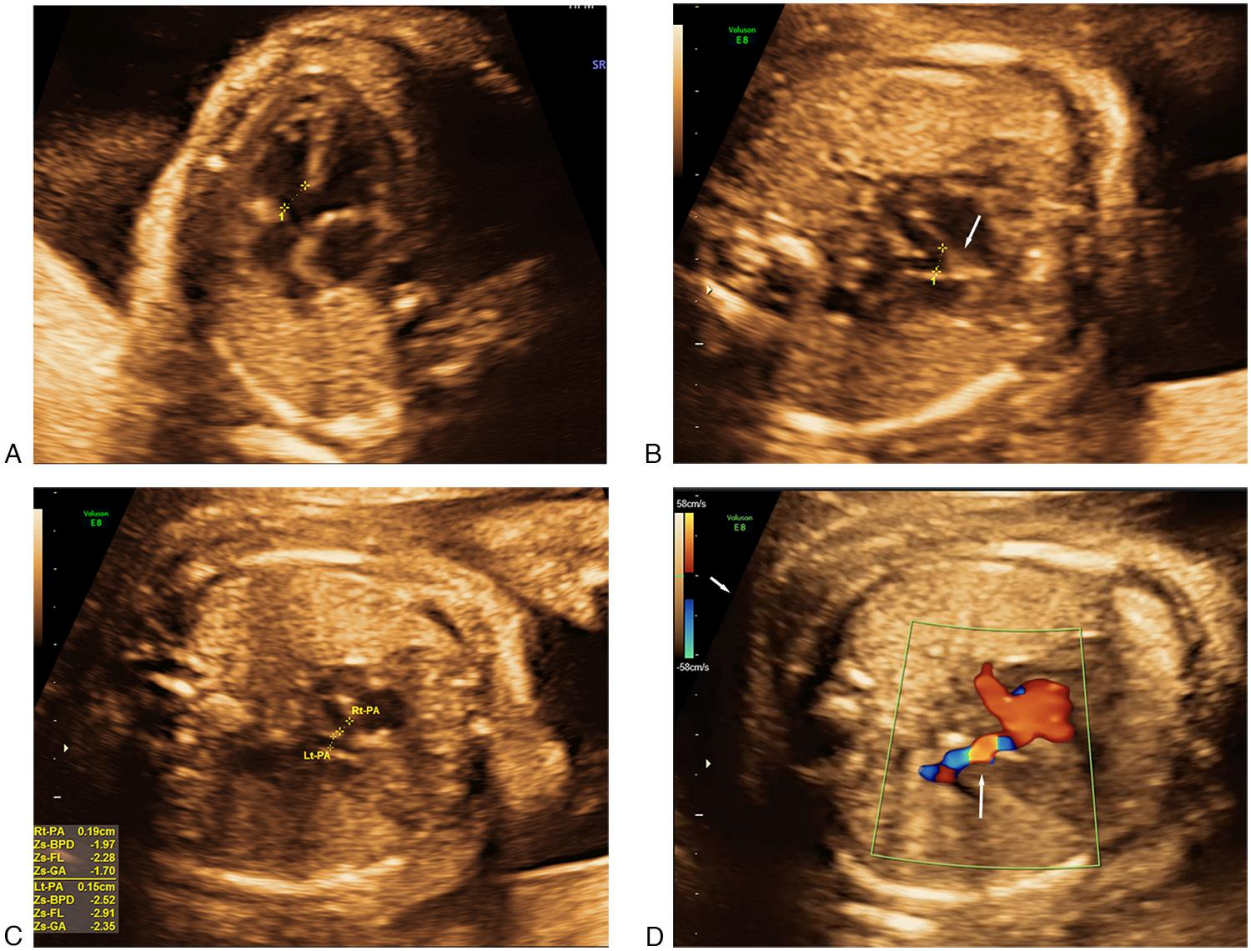
Cardiac abnormalities in fetus. **(A)** Subaortic ventricular septal defect(4.3 mm). **(B)** Absent main pulmonary artery with confluent central pulmonary arteries. **(C)** Dysplastic left and right pulmonary artery branches. **(D)** Reverse blood perfusion of ductus arteriosus.

A detailed second-trimester routine screening at 22 weeks and 5 days of gestation uncovered severe cardiac anomalies in the male fetus. Fetal echocardiography confirmed the diagnosis of PA/VSD (Figures 1A–D). The main pulmonary artery (MPA) was not identified, and only the pulmonary confluence and right and left pulmonary branches were visualized. The left and right heart exhibited symmetrical proportions, with preserved left and right ventricular function. The atrioventricular valves were within normal limits with regard to both morphology and function. Ultrasound imaging detected no other major structural anomalies. Following multidisciplinary consultation on the severity of the CHD, the necessity for complex multi-stage surgical palliation, and the uncertain long-term prognosis, the parents elected to terminate the pregnancy. The family declined postmortem examination.

## 3 Genetic analysis

Upon obtaining informed consent, fetal tissue collected post-termination and parental peripheral blood samples underwent trio whole-exome sequencing (WES). Library preparation, sequencing, read alignment, variant calling, and annotation were performed using established protocols (7). Candidate variants were validated by Sanger sequencing.

**Figure 2:**
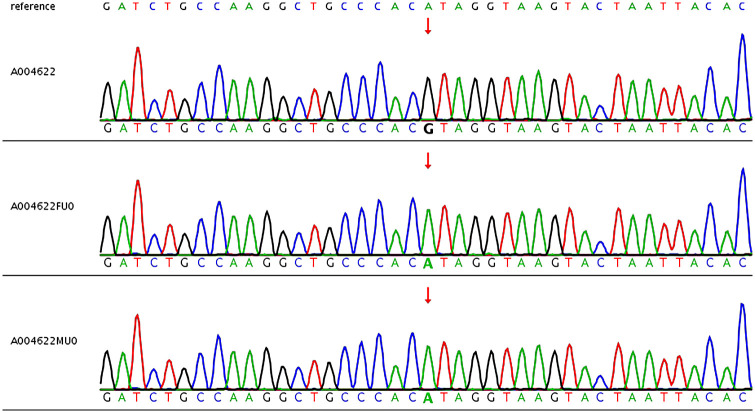
Sanger sequencing shows the *de novo* status of USP9X of c.5186A > G in the fetus.

Chromosomal abnormalities were assessed by copy number variation sequencing, and the analysis revealed a normal karyotype without evidence of aneuploidy or pathogenic copy number variations—including 22q11.2 deletion syndrome and other common chromosomal etiologies of congenital heart defects. WES identified a hemizygous missense variant in exon 33 of the *USP9X* gene (NM_001039590.3): c.5186A > G (Figure 2), predicted to result in a histidine-to-arginine substitution at codon 1729 (p.His1729Arg). This variant (dbSNP ID: rs2147230302) was absent in both parental genomes, confirming its *de novo* origin in the fetus. The potential pathogenicity of the identified *de novo* hemizygous missense variant in *USP9X*, p.His1729Arg (NM_001039590.3: c.5186A > G), was assessed using multiple lines of evidence. This variant is exceedingly rare, as it was not observed in gnomAD, a large-scale population database (8). In clinical significance databases, this variant is annotated in ClinVar (VCV001338822) as “Likely Pathogenic” based on a submission from a single clinical laboratory, which associates it with a female-restricted syndromic neurodevelopmental disorder. In support of its potential pathogenicity, multiple in silico prediction algorithms, including SIFT (9), PolyPhen-2 (10), and CADD (11) (Phred-scaled score: 24.3), predicted a deleterious effect of the p.His1729Arg substitution on protein function (Supplementary Table S1). Furthermore, the His1729 residue is localized to a conserved CHC2-type zinc-finger (ZnF) motif within the catalytic domain of *USP9X (*15). Considering the critical role of ZnF motifs in maintaining protein tertiary structure and catalytic activity, substitution of the zinc-coordinating histidine residue at this position with arginine is highly predicted to disrupt the local zinc-binding network and compromise the functional integrity of the *USP9X* catalytic domain.

The pathogenicity of the USP9X c.5186A > G variant was systematically evaluated following a Bayesian adaptation of the ACMG/AMP guidelines (12, 13). We integrated the confirmed *de novo* status (PS2), its localization to a critical region within the zinc-finger motif (PM1), absence from population frequency databases (PM2_Supporting), supportive in silico computational predictions (PP3), and emerging functional evidence indicative of splice-site alteration (PS3_Supporting). A cumulative score of 9 points was derived, leading to a Likely Pathogenic classification (Table 1). Collectively, these convergent lines of evidence strongly support this variant as the genetic etiology underlying the PA/VSD identified in the fetus.

**Table 1:** Pathogenicity classification of the USP9X variant c.5186A > G (p.His1729Arg) based on the ACMG/AMP Bayesian framework.

Evidence category	ACMG code	Strength (points)	Evidence description & application
*De novo* data	PS2	Strong (4)	The variant is confirmed as *de novo* in the affected male fetus (maternity and paternity confirmed), with no history of the variant in either parent.
Functional domains	PM1	Moderate (2)	The variant is located in the critical CHC2-type zinc-finger motif and affects a conserved zinc-coordinating histidine (His1729), which is essential for protein stability and catalytic integrity.
Functional data	PS3	Supporting (1)	*In vitro* functional studies (RNAseq) from a somatic cell line demonstrate that the c.5186A > G variant modifies a cryptic splice donor site, leading to intron retention (ClinVar SCV007017646.1). Assessed as Supporting due to the somatic nature of the source data.
Population data	PM2	Supporting (1)	The variant is absent from large population databases (gnomAD), indicating extreme rarity (PM2 downgraded to Supporting level per ClinGen SVI V1.0 recommendations).
Computational data	PP3	Supporting (1)	Multiple computational algorithms (SIFT, PolyPhen-2, CADD) predict a deleterious effect on the gene or gene product.
Total score		9 points	Calculation: 4 (PS2) + 2 (PM1) + 1 (PS3) + 1 (PM2) + 1 (PP3) = 9
Final classification		Likely pathogenic	Posterior Probability > 90% (Consistent with Likely Pathogenic range: 6–9 points)

## 4 Discussion

This case report describes for the first time a novel genotype-phenotype association between the *de novo* hemizygous *USP9X* missense variant p.His1729Arg and the severe CHD (PA/VSD) in a male fetus. While *USP9X* variants have been primarily associated with NDDs (7, 8), our finding substantially advances the understanding of *USP9X*'s pleiotropic roles beyond neurodevelopment, particularly in cardiogenesis.

Previous studies have suggested cardiac involvement in *USP9X*-related disorders. Cardiac defects are recognized features of XLID99 in females, and Agazzi et al. described a male case with a distinct *USP9X* variant (p.Met1824Val) presenting with complex CHD, including VSD and aortic arch anomalies (8).

Our case extends this emerging evidence base while distinguishing itself in several critical dimensions. First, this study establishes a mechanistic link between *USP9X* functional impairment and the specific severe phenotype of PA/VSD, characterized by complete RVOT obstruction, a cardiac feature not previously documented in *USP9X*-related cases.

Furthermore, this study establishes a specific association between the severe cardiac phenotype and *USP9X* p.His1729Arg, a variant classified as “Likely Pathogenic” in ClinVar database that was previously exclusively linked to female NDDs. This finding expands the phenotypic spectrum associated with this specific *USP9X* allele.

The biological plausibility linking *USP9X* dysfunction to PA/VSD is strongly supported by its role as a deubiquitinase critical for early embryonic cardiogenesis (14); Complete loss-of-function of *USP9X* is presumably embryonic lethal in males. Surviving males typically carry hypomorphic alleles that cause partial functional impairment (4, 5) The reported p.His1729Arg variant localizes to a zinc-coordinating residue within a ZnF motif, strongly predicting disruption of the zinc-binding network and functional impairment. *USP9X* has been shown to physically interact with SMAD4 (15), a central transcriptional mediator of the TGF-*β* and BMP signaling pathways. These pathways are critical for multiple cardiogenic processes, including cardiac neural crest cell migration, outflow tract septation, and cardiac valve formation—developmental events that are fundamentally disrupted in PA/VSD (15). Dysregulation of TGF-*β*/BMP signaling is a well-documented genetic cause of various CHDs (15). Notably, *USP9X* evades X-chromosome inactivation, a feature that renders embryonic developmental processes exquisitely sensitive to gene dosage fluctuations. Therefore, partial loss of *USP9X* function resulting from the hemizygous p.His1729Arg mutation, likely disrupted critical signaling for cardiac development, consequently leading to the observed PA/VSD in this male fetus. Although NDDs could not be evaluated *in utero*, it is biologically plausible that neurodevelopmental sequelae would have emerged postnatally had the pregnancy proceeded, given the well-established role of *USP9X*.

Expanding on the functional evidence outlined in our genetic analysis, it is noteworthy that beyond the predicted disruption of the zinc-finger protein domain, emerging functional data suggests a potential impact on transcript processing. An entry in the ClinVar database (Accession: SCV007017646.1), based on RNA-seq analysis, indicates that the c.5186A > G (p.His1729Arg) variant may alter an exonic cryptic donor splice site, leading to significant intron inclusion (*p* = 0.0004). This finding suggests the variant may exert a deleterious effect via a dual mechanism: impairment of the protein's catalytic structure and induction of aberrant splicing. Both mechanisms converge to elicit a loss-of-function effect, which is consistent with the well-established haploinsufficiency mechanism underlying USP9X-related disorders.

This case underscores the critical need to include *USP9X* in the genetic differential diagnosis of severe, idiopathic CHD, particularly in male patients or those with syndromic features, even when NDDs are not the primary prenatal presentation. It underscores the critical utility of comprehensive genomic profiling, such as WES, in disentangling novel gene-disease associations within complex developmental disorders. The finding that a *USP9X* variant previously linked exclusively to female NDDs manifests as severe CHD in a male fetus underscores the gene's phenotypic pleiotropy and highlights potential sex-specific differences in variant expression.

Study limitations include its single-case design and the absence of post-mortem histological analysis or mechanistic validationto to directly assess the impact of p.His1729Arg variant on *USP9X*-mediated signaling and cardiac morphogenesis. However, while no specific animal models were generated for the present study, our systematic Bayesian analysis (presented in the Results and Table 1) yielded a cumulative score of 9, which firmly classifies this variant as Likely Pathogenic. This designation is supported by multiple independent lines of evidence, including confirmed *de novo* status, a critical structural impact on the zinc-finger domain, and emerging RNA-seq data indicative of splicing defects.

## 5 Conclusion

We herein report the first case establishing a *de novo* p.His1729Arg missense mutation in *USP9X* as a cause of PA/VSD in a male fetus. This discovery significantly expands the clinical spectrum of this “Likely Pathogenic” variant, and reinforces *USP9X*'s critical role in human cardiogenesis, putatively via modulation of essential developmental pathways like TGF-*β*/BMP signaling. Our findings underscore the clinical imperative for comprehensive cardiac assessment in individuals harboring pathogenic *USP9X* variants. Concurrently, we advocate for the inclusion of *USP9X* in multi-gene diagnostic panels for severe CHD. Future investigations, including large-cohort studies and mechanistic analyses, are essential to fully characterize *USP9X* genotype-phenotype correlations and dissect the pathomechanisms underlying its role in cardiac development.

## Data Availability

The original contributions presented in the study are included in the article/supplementary material, further inquiries can be directed to the corresponding authors.
